# Environmental Alkylphenols Modulate Cytokine Expression in Plasmacytoid Dendritic Cells

**DOI:** 10.1371/journal.pone.0073534

**Published:** 2013-09-11

**Authors:** Chih-Hsing Hung, San-Nan Yang, Ya-Fang Wang, Wei-Ting Liao, Po-Lin Kuo, Eing-Mei Tsai, Chin-Lai Lee, Yu-Shen Chao, Hsin-Su Yu, Shau-Ku Huang, Jau-Ling Suen

**Affiliations:** 1 Department of Pediatrics, Faculty of Pediatrics, Kaohsiung Medical University, Kaohsiung, Taiwan; 2 Graduate Institute of Medicine, College of Medicine, Kaohsiung Medical University, Kaohsiung, Taiwan; 3 Department of Pediatrics, Kaohsiung Medical University Hospital, Kaohsiung, Taiwan; 4 Center of Excellence For Environmental Medicine, Kaohsiung Medical University, Kaohsiung, Taiwan; 5 Departments of Medical Laboratory Science and Biotechnology, National Cheng Kung University, Tainan, Taiwan; 6 Center of Infectious Disease and Signaling Research, Medical College, National Cheng Kung University, Tainan, Taiwan; 7 Department of Biotechnology, College of Life Science, Kaohsiung Medical University, Kaohsiung, Taiwan; 8 Institute of Clinical Medicine, Kaohsiung Medical University, Kaohsiung, Taiwan; 9 Division of Environmental Health and Occupational Medicine, National Health Research Institutes, Miaoli County, Taiwan; 10 Johns Hopkins Asthma and Allergy Center, School of Medicine, Johns Hopkins University, Baltimore, Maryland, United States of America; National Jewish Health and University of Colorado School of Medicine, United States of America

## Abstract

**Background:**

Alkylphenols, such as nonylphenol (NP) and 4-octylphenol (4-OP), have the potential to disturb immune system due to their weak estrogen-like activity, an effect with potential serious public health impact due to the worldwide distribution of these substances. Plasmacytoid dendritic cells (PDCs) can secrete large amounts of type I IFNs and are critical in immune regulation. However, there has been limited study about the influence of alkylphenols on the function of pDCs.

**Objective:**

The aim of this study was to examine the effect of alkylphenols on pDC functions *in vitro* and *in vivo* and then further explored the involved signaling pathways and epigenetic changes.

**Methods:**

Circulating pDCs from human peripheral blood mononuclear cells were treated with alkylphenols with or without CpG stimulation. Alkylphenol-associated cytokine responses, signaling events, histone modifications and viral activity were further examined. In NP-exposed mice, the effect of NP on splenic pDC function and allergic lung inflammation were also assessed.

**Results:**

The results showed that NP increased the expression of TNF-α, but suppressed IL-10 production in the range of physiological doses, concomitant with activation of the MKK3/6-p38 signaling pathway and enhanced levels of acetylated histone 3 as well as histone 4 at the *TNFA* gene locus. Further, in CpG-stimulated pDCs, NP suppressed type I IFNs production, associated with down-regulation of IRF-7 and MKK1/2-ERK-Elk-1 pathways and led to the impaired anti-enterovirus 71 activity *in vitro*. Additionally, splenic pDCs from NP-exposed mice showed similar cytokine changes upon CpG stimulation under conditions relevant to route and level of exposure in humans. NP treatment also enhanced allergic lung inflammation *in vivo*.

**Conclusion:**

Alkylphenols may influence pDCs’ functions via their abilities to induce expression of a pro-inflammatory cytokine, TNF-α, and to suppress regulatory cytokines, including IL-10, IFN-α and IFN-β, suggesting the potential impact of endocrine disrupting chemicals on immune regulation.

## Introduction

Endocrine disrupting chemicals (EDCs) have been ubiquitous in the environment throughout the years after World War II, concomitant with an increase in the prevalence of both allergic and autoimmune diseases [1]. Allergic diseases and autoimmune diseases are chronic inflammatory diseases characterized by dys-regulated immunity against allergens or self-antigens, respectively. EDCs may have various undesirable effects on human health due to their hormone like activities [2]; however, the exact regulatory mechanisms remain to be elucidated.

Among EDCs, alkylphenol polyethoxylates are widely used as non-ionic surfactants in household applications, industrial and personal care products. Previous studies have shown that nonylphenol (NP) and 4-octylphenol (4-OP), two major degradation products of alkylphenol polyethoxylates, have weak estrogenic activities [3] and may disturb endocrine system function [4]. Alkylphenols have been shown to undergo significant bioaccumulation due to their lipophilic properties [5]. They also have been detected in water, foods and breast milk [6]. As estrogens play a significant role in the development and regulation of immune cells [7], NP and 4-OP may be associated with immune disorder diseases, such as allergic diseases and autoimmune diseases [8].

Dendritic cells (DCs) have emerged as major antigen-presenting cells with a critical role in initiation and regulation of adaptive immune responses. Plasmacytoid DCs (PDCs) are important for the regulation of immunity via secreting cytokines, such as IFN-α, IFN-β, IL-10 and TNF-α [9]. IFN-α and IFN-β have direct anti-viral activity and other indirect biological effects, including Th1 differentiation, cytotoxic T lymphocyte differentiation, natural killer cell activity and upregulation of costimulatory molecules on conventional DCs [10]. IFN-α has the largest number of family members and plays an important role in the defense against pathogens. IRF-7 plays a critical role for IFN-α expression in pDCs, and some infections impair the ability of pDCs to produce IFN-α by blocking the TLR9-IRF-7-IFN-α signaling pathway [11]. In addition, IL-10 has anti-inflammatory properties and modulates expression of cytokines, soluble mediators and cell surface molecules by macrophages and DCs [12]. TNF-α is significantly involved in the pathogenesis of allergic diseases and autoimmune diseases. Thus, it is regarded as a target of treatment in asthma, SLE and rheumatoid arthritis [13].

It is largely unknown, however, as to whether alkylphenols have any effect on pDCs to modulate immune function. Considering the importance of pDCs in various disease contexts, the goal of this study was to explore the functional influence of alkylphenols on pDCs *in vitro* and *in vivo*. We have examined the *in vitro* effects of two akylphenols, NP and 4-OP, on the expression of three regulatory cytokines in human pDCs, and have provided, for the first time, evidence supporting the influence of EDCs on pDC’s function *in vivo*.

## Materials and Methods

### Isolation and Treatment of Human PDCs

The study of human subjects was approved by the Institutional Review Board of Kaohsiung Medical University, Taiwan (KMUH-IRB-990392). PBMCs were isolated from 12 volunteer subjects after obtaining written informed consent, and circulating pDCs were magnetically sorted by centrifugation with BDCA-4 cell isolation kits (Miltenyi Biotec). The purity of isolated pDCs was at least >90%. Purified pDCs were treated with DMSO (vehicle control), NP, 4-OP (Tokyo Chemical Industry), or 17β-estradiol (E2) (Sigma-Aldrich) for 48 h. For type I IFN responses, pDCs were also treated with NP for 2 h and then stimulated with 10 µg/ml of CpG-oligodeoxynucleotide 2216 (CpG-2216) with or without IL-3 (10 ng/ml) for 48 h. To examine the involved receptors or signaling pathways, pDCs were pre-treated with anacardic acid (histone acetyltransferase inhibitor, Calbiochem), ICI182,780 [estrogen receptor (ER) antagonist], and three MAPK inhibitors (PD98059, SB203580 and SP600125, Sigma-Aldrich) 1 h before alkylphenol treatment. The supernatant was further assessed regarding the cytokine profile. After 48 h MAPK inhibitor treatment, the viabilities of pDCs were at least 95% as analyzed by WST-1 viability kit (ScienCell) (data not shown).

### Western Blotting

After treatment for 2 h with or without NP, the cells were stimulated with or without CpG-2216 (10 µg/ml) plus IL-3 (10 ng/ml) and lysed in lysis buffer 1 h later. Then equal amounts of cell lysates were analyzed by western blotting with anti-IRF-7, anti-histone 3 (H3) (Cell Signaling Technology), anti-β-actin, anti-MEK-1/2, anti-phospho-MEK1/2, anti-MKK-3/6, anti-phospho-MKK3/6, anti-MAPK (p38, ERK or JNK) and anti-phospho-MAPK (pp38, pERK or pJNK) antibodies (Santa Cruz Biotechnology). For the IRF-7 study, cell lysate was collected 6 h after CpG and IL-3 stimulation. In some experiments, nuclear/cytoplasmic fractionation was carried out according to the manufacturer’s instruction (Novagen).

### ERK Kinase Activity Assay

After treatment, the cell lysate was immunoprecipitated with anti-pERK Ab and pERK activity was measured by nonradioactive ERK assay kit (Cell Signaling Technology). Elk-1 was used as a substrate for ERK MAPK assay *in vitro*. Elk-1 phosphorylation was detected by anti-pElk-1 Ab (Cell Signaling Technology) in western blotting assay.

### ELISA for Determination of the Cytokine Levels

The production of cytokines in the culture supernatants was determined for IFN-α, IFN-β (PBL InterferonSource)??IL-10 and TNF-α by ELISA (R&D system).

### Chromatin Immunoprecipitation (ChIP) Assay

ChIP assay was performed as previously described [14]. Briefly, pDCs in each condition was fixed with 1% formaldehyde for 10 minutes at room temperature. The lysed and sonicated pDCs were incubated with acetylated H3, acetylated histone 4 (H4) (Upstate Biotechnology, Waltham, MA), or rabbit anti-BSA (Sigma-Aldrich, St. Louis, MO) as a control at 4°C overnight. The released DNA fragments were subjected to PCR or real time PCR detection of the *TNFA* promoter and enhancers. Input DNA was used as a positive control. Seven pairs of primers were used to analyze the corresponding regions of the *TNFA* promoter and introns. The numbers in parentheses after the TNF label represented PCR amplification sites. They include the various *TNFA* promoter regions relative to the transcription start site [15], [16]: TNF1 (+99/−42); TNF2 (+32/−119), TNF3 (–100/−250), TNF4 (–195/−345), 1417 (+1391/+1431), +720 (+762/+799) and −1700 (−1694/−1758). The relative density of PCR product from each ChIP sample was normalized to that from corresponding input DNA. Fold enrichment is defined as the normalized ChIP signal of NP-treated cells versus that of vehicle-treated cells. Differential DNA binding of Acetyl H3 or H4 in treated pDCs was calculated by 2^−ΔΔCT^method.

### RT-PCR

Purified human pDCs were treated with 10^−7^ M of NP or vehicle without CpG stimulation for 6 h. Total RNA was isolated by TRIzol kit and converted to cDNA by the SuperScript II kit (Invitrogen) using specific primer pairs. *TNFA* gene expression was normalized to β-actin mRNA copies from the same sample.

### Mice

All animal work was approved by the IACUC at the Kaohsiung Medical University (Permit Number: 99032). Female BALB/cByJNarl mice were obtained from National Laboratory Animal Center and maintained by the Animal Center of Kaohsiung Medical University in a pathogen-free facility.

### In Vivo Treatment and Assessment of PDC Function and Airway Inflammation

BALB/c mice at 6–8 weeks of age were orally fed 5 µg NP/kg body weight (BW)/day or corn oil alone (negative control, NC group) for 10 days. Splenocytes were further negatively selected for pDCs using a commercial kit (Miltenyi Biotec). The phenotype and purity of viable splenic pDCs (90–95%) was analyzed by LSRII. The purified pDCs from two independent experiments were treated with CpG-D19 (3 µM) for IFN-α, CpG-1668 (3 µM) for TNF-α or IL-12, and R848 (100 nM) for IL-12.

For the lung inflammation model, BALB/c mice at 4 weeks of age were intraperitoneally received PBS, OVA (10 µg/mice) or OVA plus NP (50 µg/kg BW) emulsified with Al(OH)_3_ on day 0. Three weeks later, all groups of mice were received three daily 3% OVA aerosol challenges. The next day after the last challenge, cells in bronchoalveolar lavage fluid were analyzed using flow cytometry [17]. Cells in BALF were stained with PE-Cy7-anti-CD11c and FITC-anti-I-A^d^/I-E^d^ (DCs/macrophages), PE-anti-CCR3 (eosinophils), APC-anti-CD3 and anti-CD19 (lymphocytes).

### In Vitro Cytopathic Effect (CPE) Protection Assay

The antiviral activities of type I IFNs were determined by *in vitro* CPE protection assay. Human rhabdomyosarcoma (RD) cells were plated on 24-well plates and grown overnight to obtain >80% confluence. Cells were pretreated with recombinant human IFN-α or supernatants from treated pDCs for 6 h and then infected with enterovirus 71 (EV71)/Tainan/4643/98 at a multiplicity of infection of 0.001. When 90% of the cells in the non-treated wells had CPE (44 h post-infection), the supernatants were collected for viral titration and the cells were fixed with 10% formaldehyde and stained with 0.5% crystal violet. The viral titers were determined by plaque assay. Briefly, confluent monolayer of RD cells was prepared in 12-well plates and incubated overnight. Cells were infected with 10-fold serial dilutions of viruses, overlaid with 0.75% methylcellulose for 3 days before the plaques were visualized using staining with 0.5% crystal violet.

### Statistical Analysis

All data are presented as mean ± SD. Change in cytokines at different doses of alkylphenol was analyzed by using the Wilcoxon signed rank test. Differences between murine experimental and control groups were analyzed by using the nonparametric Mann-Whitney U test. A *P* value <0.05 was considered indicative of significant between group differences.

## Results

### NP Enhanced the Proinflammatory Activity of PDCs via MKK 3/6-p38 MAPK Pathway

**Figure 1:**
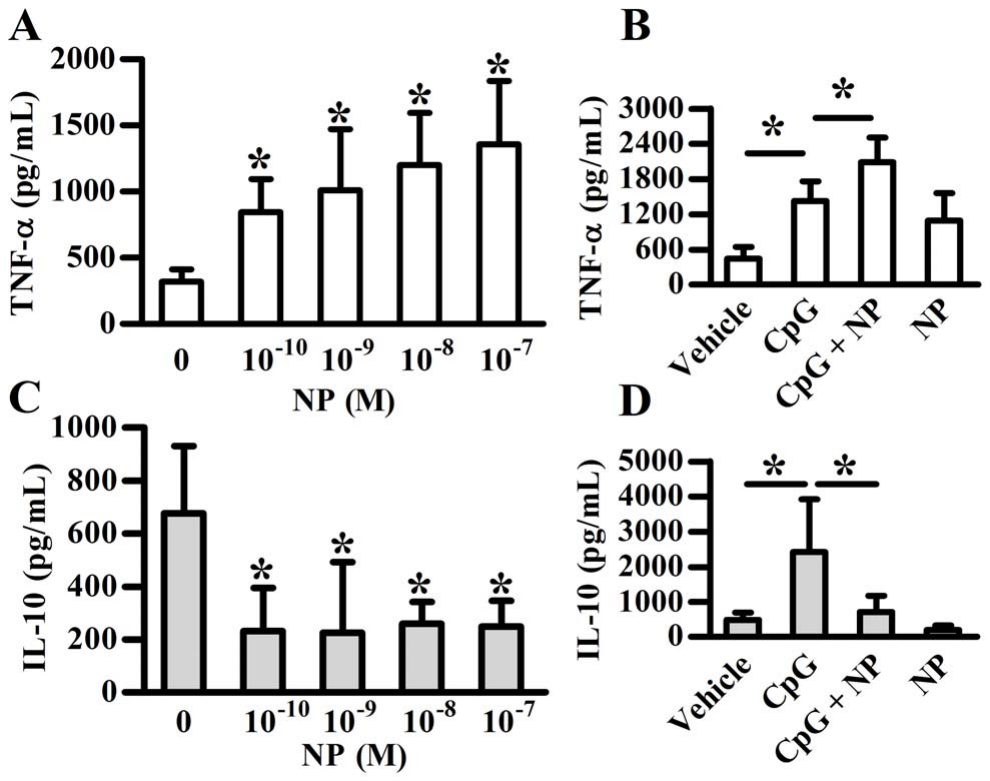
NP enhances TNF-α but inhibits IL-10 production of human pDCs. Purified human plasmacytoid dendritic cells (pDCs) were treated with various concentrations of NP (nonylphenol) with or without CpG stimulation for 48 h. The supernatant was collected for TNF-α (**A**, **B**) or IL-10 (**C**, **D**) detection using ELISA method. Data show means ± SD of five independent experiments. The NP concentration used in *B* and *D* was 10^−7^ M. *p<0.05 was considered significant versus corresponding control.

To examine whether NP has a direct impact on pDC’s function, the expression of cytokines, TNF-α, IL-10, IFN-α and IFN-β in pDCs treated with or without different doses of NP was analyzed. As the K_ D_ of NP for ERα is around 0.05% of the affinity of E2 (10^−10^ M) [3], [18], the calculated K_ D_ of NP for ERα is around 2×10^−7^ M. In addition, due to the lipophilic property, the level of NP in adipose tissue is 10-fold higher than that in plasma [19]; that is around 10^−8^ and 10^−7^ M. Thus, we initially tested the effect of NP in the range of 10^−10^ M to 10^−7^ M. The results showed that NP significantly enhanced TNF-α (Fig. 1A) expression in a dose-response manner, but suppressed the level of IL-10 even at the dose of 10^−10^ M (Fig. 1C). Upon CpG stimulation, NP still effectively increased the TNF-α (Fig. 1B) and inhibited the IL-10 (Fig. 1D) production. In addition, another important alkylphenol, 4-OP, also significantly enhanced TNF-α and inhibited IL-10 production of human pDCs (Figs. S1A and S1B).

**Figure 2:**
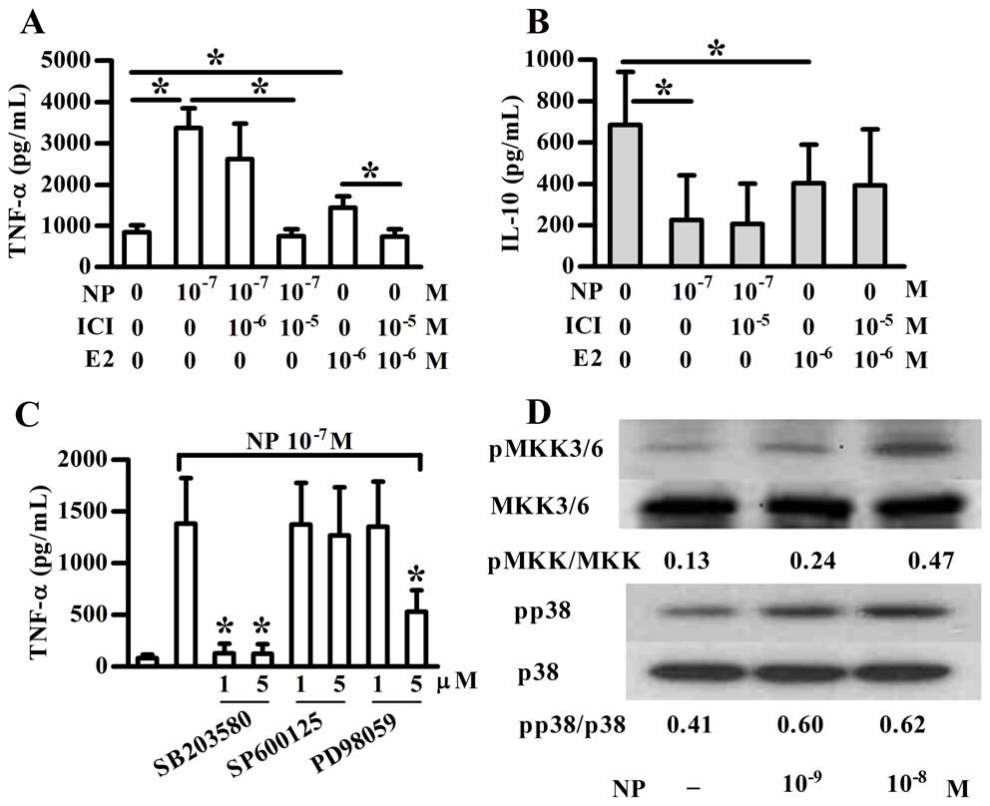
The effect of NP on cytokine expression of pDCs is mediated, in part, via estrogen receptor. Purified human plasmacytoid dendritic cells (pDCs) were treated with nonylphenol (NP) or 17β-estradiol (E2) at indicated concentrations for 48 h. ICI182,780 or MAPK inhibitor was added as indicated concentration 1 h before NP or E2 treatment. The supernatant was collected for TNF-α (**A**, **C**) or IL-10 (**B**) detection using ELISA method. Data show means ± SD of five independent experiments. *p<0.05 was considered significant versus control. (**D**) After 1 h NP treatment, total cell lysate was analyzed by western blotting. Data are from one representative of three independent experiments.

To examine the role of ER in mediating the NP effect, pDCs were cultured in the presence or absence of NP or E2, and the levels of TNF-α were analyzed. As shown in Fig. 2A, purified pDCs secreted more TNF-α in response to NP or E2 stimulation. Both NP-induced and E2-induced TNF-α secretion were inhibited by ICI182,780, an ER antagonist. However, the effect of E2 or ICI182,780 on pDC cytokine expression was only observed at relative high concentrations, suggesting that NP induced TNF-α expression in pDCs was not only mediated by ERs, but also other receptors. Furthermore, ICI182,780 could not reverse the suppressive effect of NP on IL-10 expression in pDCs, suggesting another mechanism(s) involved in NP suppressed IL-10 expression in pDCs (Fig. 2B).

Next, as the MAPK signaling pathway is involved in various cellular functions and is highly conserved in many types of immune cells, we first examined whether the MAPK signaling pathway regulated TNF-α production in pDCs. The MAPK family includes three subgroups – JNK, ERK, and p38 MAPK. Their corresponding specific inhibitors were used to pre-treat purified human pDCs before NP treatment. Fig. 2C showed that SB203580 (p38 MAPK inhibitor) significantly suppressed NP-induced TNF-α expression while a higher dose of PD98059 (ERK inhibitor) was required to inhibit NP-induced TNF-α production of pDCs. In addition, the level of pp38 was increased in NP-treated cells (Fig. 2D). MKK3/6 is the upstream signaling molecule of p38. NP also increased levels of pMKK3/6, suggesting that NP induced TNF-α expression may be through the MKK3/6-p38 MAPK pathway. We also examined the JNK and ERK pathways; however, NP had no direct effect on these two kinases.

### NP Mediated Histone Acetylation at the TNFA Gene Locus

**Figure 3:**
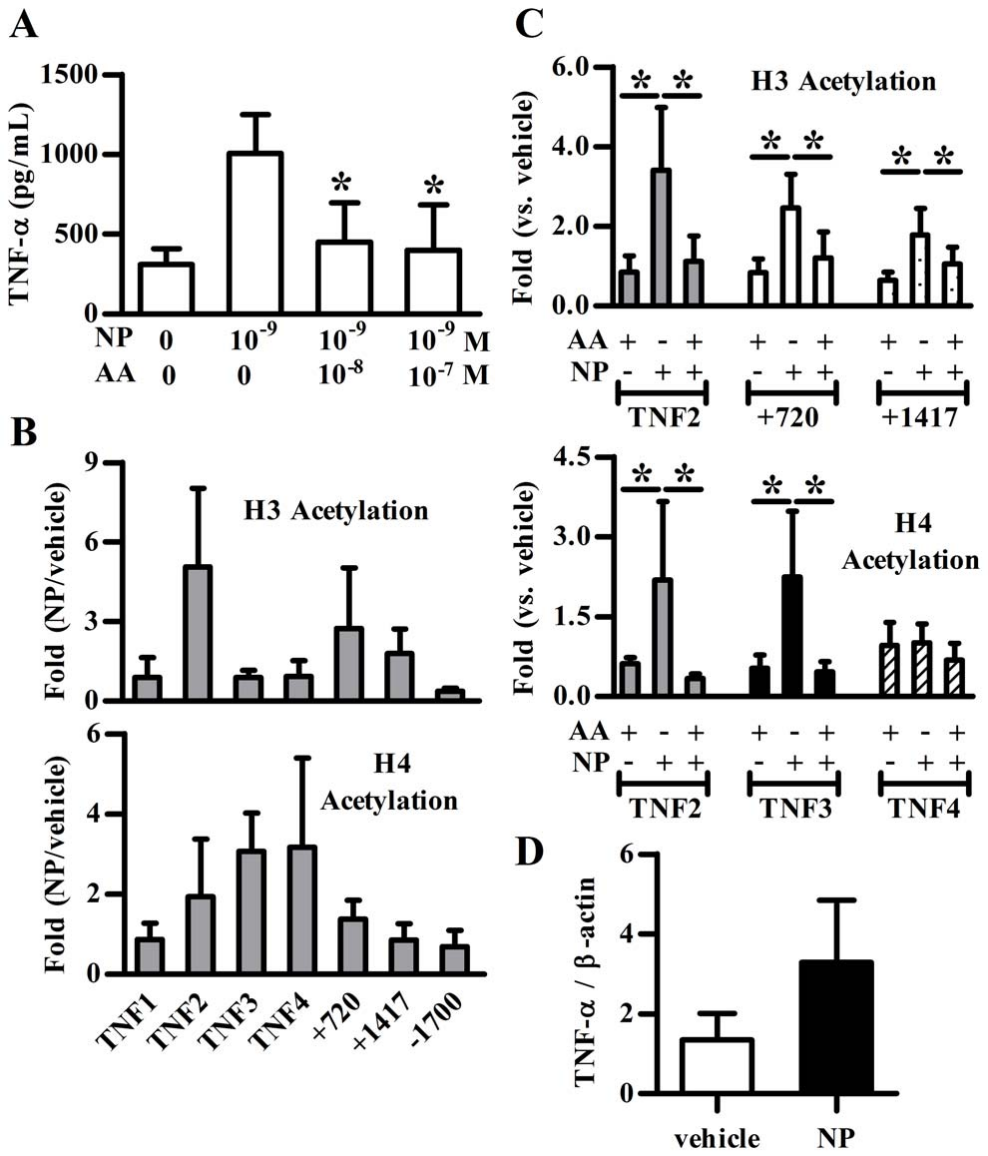
The involvement of histone acetylation in NP effect on TNF-α expression. (**A**) The levels of TNF-α expression in nonylphenol (NP)-treated plasmacytoid dendritic cells (pDCs) in the presence or absence of different doses of anacardic acid (AA, a histone acetyltransferase inhibitor). *p<0.05 was considered significant versus without inhibitor treatment. (**B, C**) Purified pDCs treated with vehicle or NP (10^−8^ M) for 1 h were subjected to chromatin immunoprecipitation (ChIP) assay. Anacardic acid was added 1 h before NP treatment. ChIP analyses of the relative levels of acetylated histone 3 (H3) and acetylated histone 4 (H4) at the *TNFA* gene locus using PCR (**B**) or real time PCR (**C**) method. It includes the following subregions relative to the transcription start site: TNF1 (+99 to –42); TNF2 (+32 to –119), TNF3 (–100 to –250), TNF4 (–195 to –345), +720 and +1417 and –1700. Results are shown as fold enrichment (mean ± SD of five individual study subjects). (**D**) Purified human pDCs were treated with 10^−7^ M of NP or vehicle without CpG stimulation for 6 h. The cells were harvested for RNA extraction, and RT-PCR was performed for analyzing TNF-α mRNA expression. Data show density ratio (TNF-α/β-actin, mean ± SD) of three individual study subjects.

As NP alone was shown to be able to induce TNF-α expression in pDCs, we sought to examine the underlying mechanism. Histone acetylation has been reported to influence TNF-α expression [15], [16]. To examine whether the *TNFA* gene locus underwent histone modification in alkylphenol-treated pDCs, ChIP analysis of NP-treated pDCs was performed, using PCR primers corresponding to five overlapping subregions (–1700 and TNF1-4, covering the region between –345 and +99) in the promoter and three intronic regions (+720 and +1417), of the *TNFA* gene. As shown in Fig. 3A and Fig. S1C, anacardic acid, a histone acetyltransferase inhibitor, suppressed NP or 4-OP-induced TNF-α expression in pDCs suggesting histone acetylation plays an important role in NP- and OP-induced TNF-α expression in pDCs. Furthermore, as compared to those found in vehicle control, significant histone modifications at the *TNFA* gene locus were observed in NP-treated pDCs. As seen in Fig. 3B, NP induced TNF-α expression was accompanied by increased histone H3 acetylation at the proximal promoter subregion (TNF2) and intron sequence (+720 and +1417) of the *TNFA* gene. NP enhanced histone H4 acetylation at the proximal promoter subregions (TNF2, TNF3 and TNF4) of the *TNFA* gene. Furthermore, anacardic acid treatment did significantly inhibit NP-induced histone H3 acetylation at the TNF2, +720 and +1417 subregions and NP-mediated histone H4 acetylation at TNF2 and TNF3, except TNF4 subregions (Fig. 3C). In addition, NP treatment increased TNF-α mRNA expression in human pDCs (Fig. 3D). These findings suggest that the effects of NP on TNF-α production of pDCs are associated with differential histone acetylations.

### NP Suppressed CpG-induced Type I IFN Expression via IRF-7 and ERK Pathway in PDCs

**Figure 4:**
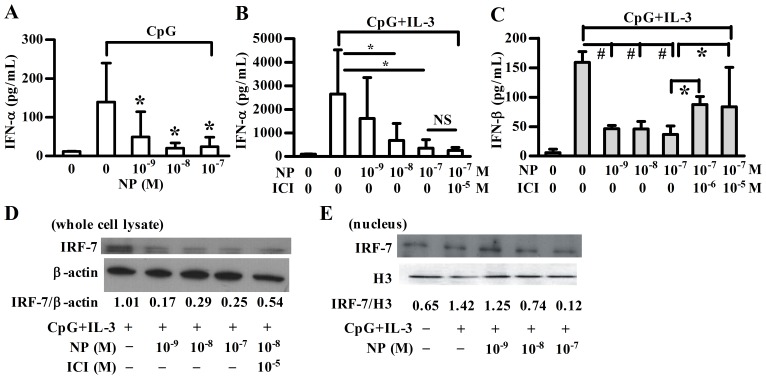
NP inhibits CpG-induced type I IFN production and IRF-7 expression in human pDCs. Purified human plasmacytoid dendritic cells (pDCs) were pre-treated with nonylphenol (NP) at various concentrations for 2 h and then stimulated with CpG (**A**) or CpG plus IL-3 (**B**, **C**) for 48 h. An estrogen receptor antagonist (ICI182,780) was added 1 h before NP treatment. The supernatant was collected for IFN-α (**A**, **B**) and IFN-β (**C**) detection using ELISA method. Results show means ± SD of five independent experiments. * or #, *p*<0.05 was considered significant versus corresponding untreated cells. (**D**) After 2 h NP treatment, purified pDCs were stimulated with or without CpG plus IL-3 for another 6 h for total IRF-7 expression. (**E**) Nuclear cytoplasmic separation was performed and nuclear protein (IRF7 or internal control Histone 3) was analyzed by Western blotting. Data are from one representative of three independent experiments.

PDCs are highly specialized immune cells that produce large amounts of type I IFNs in response to viral infection via, in part, TLR7 and TLR9 [11]. To investigate whether NP has any functional effect on TLR9 ligand (CpG)-induced expression of IFNs, pDCs were treated with varying concentrations of NP and CpG oligonucleotides, and the levels of both IFN-α and IFN-β were determined. As shown in Figs. 4A and 4B, NP suppressed CpG or CpG plus IL-3-induced IFN-α production in pDCs in a dose-dependent manner. In addition, NP also significantly inhibited IFN-β production of pDCs in response to CpG plus IL-3 stimulation (Fig. 4C). Also, 4-OP significantly inhibited type I IFN production in pDCs in response to CpG stimulation (Figs. S2A and S2B).

As estrogen can enhance the response of pDCs to CpG [20], ICI182,780 was used to block the signaling mediated by ERα and ERβ. Data showed that ICI182,780 partially reversed the inhibitory effect of NP on IFN-β production (Fig. 4C), but it did not influence the NP effect on IFN-α secretion (Fig. 4B). These data suggest that the suppressive effect of NP on IFN-α and IFN-β expression in pDCs was through an ER independent and dependent mechanism, respectively.

As IRF-3 and IRF-7 are important transcription factors for type I IFN expression [11], we next examined whether NP affected their expression. As shown in Fig. S3A, human pDCs constitutively expressed both IRF-3 and IRF-7, and upon CpG plus IL-3 stimulation, pDCs significantly expressed IRF-7, but not IRF-3. After NP treatment, the level of total IRF7 expression was significantly decreased and was partially reversed by ICI182,780 (Fig. 4D). Although the level of total IRF7 did not decrease in a dose-dependent manner, the nuclear IRF7 expression did gradually decrease along with the 10-fold increase of NP concentrations as shown in Fig. 4E. Additionally, the transcription of the IFNA2, IFNA10 and IFNA21 genes was reduced in response to NP treatment (Fig. S3B).

**Figure 5:**
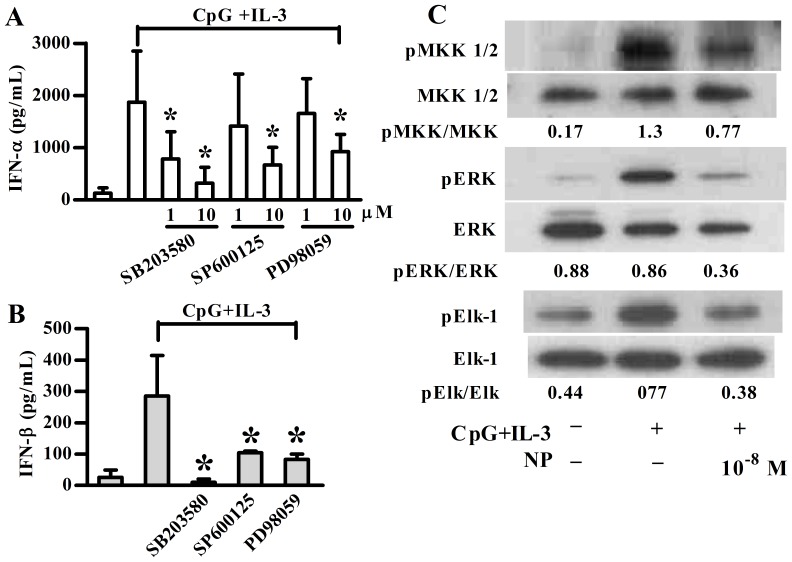
NP treatment affects the CpG-induced MAPK signaling in human pDCs. MAPK inhibitors (10 µM) were added 1 h before plasmacytoid dendritic cells (pDCs) were stimulated with CpG plus IL-3 for 48 h. The supernatant was collected for IFN-α (**A**) and IFN-β (**B**) detection using ELISA method. Results show means ± SD of five independent experiments. *p<0.05 was considered significant versus untreated cells. (**C**) After 2 h nonylphenol (NP) treatment, purified pDCs were stimulated with CpG plus IL-3 for another 1 h. The total cell lysate was analyzed by western blotting with different signaling molecules as indicated. After pERK immunoprecipitation, pERK kinase activity was assessed using Elk-1 as substrate. Data are from one representative of three independent experiments.

Next, we examined whether MAPK pathways are involved in the NP effect on type I IFN production. First, we found that all the three MAPK members are involved in type I IFN production of pDCs (Figs. 5A and 5B). We then found that NP suppressed CpG plus IL-3-induced pMEK1/2 and pERK protein expression in pDCs (Fig. 5C). Using an ERK kinase assay, the pERK activity was determined by the level of pElk-1. As shown in Fig. 5C, the pERK activity was also inhibited by NP treatment. However, NP-treated pDCs demonstrated no effect on CpG plus IL-3-induced JNK and p38 MAPK in pDCs (data not shown). These data suggest that NP is able to influence the regulatory pathway leading to CpG plus IL-3-induced type I IFN expression, involving IRF-7 and ERK.

As previous studies have demonstrated that MAPK signaling in human monocyte-derived DCs plays an important role in DC maturation [21], we also checked the phenotype of pDCs during the culture time period. We did not observe any significant difference in the expression levels of DC markers, including CD40, CD80, CD86 and HLA-DR, as analyzed by flow cytometry (data not shown).

### NP Treatment Impaired Type I IFNs-Dependent Anti-Viral Activity in Vitro

**Figure 6:**
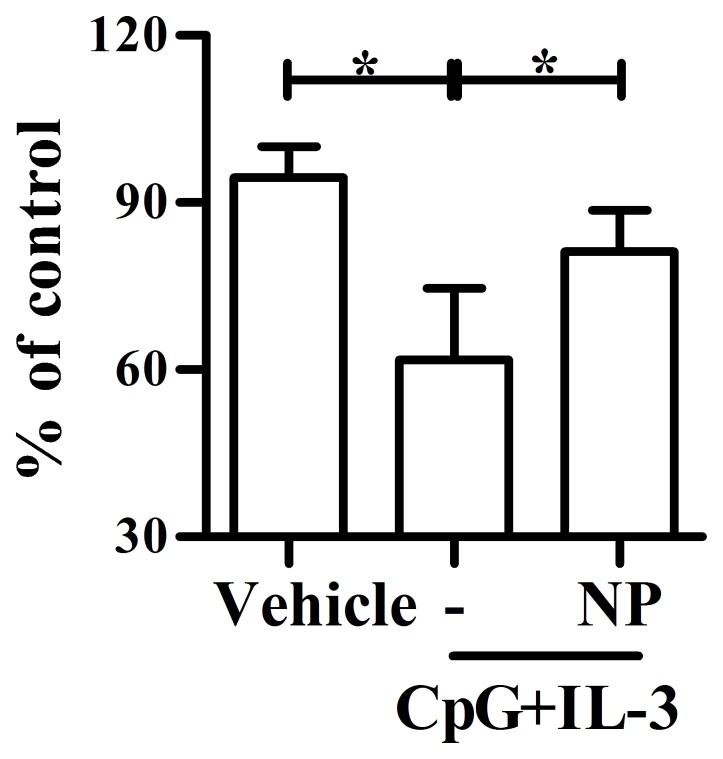
NP treatment impairs IFN-α-dependent protection against enterovirus 71 infection *in vitro*. Purified human plasmacytoid dendritic cells (pDCs) from 4 healthy donors were treated with vehicle, nonylphenol (NP) (10^−7^ M) and CpG plus IL-3 as described in Fig. 4B. Human rhabdomyosarcoma (RD) cells were treated with the supernatants from treated human pDCs for 6 h and then infected with enterovirus 71 (EV71)/Tainan/4643/98 at multiplicity of infection of 0.001. After 44 h, the supernatants were collected for viral titration. The viral titers were determined by plaque assay using RD cells. The mean viral titer of non-treated RD cells was 2.85×10^7^ PFU/ml and set as 100%. Results are shown as relative viral titer (%) of supernatant from treated pDCs compared to non-treated RD cells. *, *p*<0.05 compared with control using nonparametric Mann-Whitney *U* test.

As type I IFNs play an important role in controlling EV71 infection and replication [22], we examined whether NP treatment impaired the type I IFNs-dependent protection against EV71-induced cytopathology. As shown in Fig. 6, EV71 infection resulted in an obvious CPE in non-treated RD cells and the viral titer determined by plaque assay was around 2.85×10^7^ PFU/ml. As human pDCs treated with vehicle in absence of CpG plus IL-3 produced little Type I IFNs, their viral titer was similar to that of non-treated cells; CpG plus IL-3 stimulated human pDCs to secrete a certain amount of IFN-α (1368–6996 pg/ml in these four donors) and their viral titers were significantly decreased as compared with their corresponding vehicle controls. In contrast, NP treatment diminished the levels of IFN-α with a corresponding significant increase in viral titers in contrast to CpG plus IL-3 treatment. These results suggested that NP exposure could impair pDC-dependent antiviral immune responses.

### Oral Exposure to NP Modulated the Cytokine Pattern of Splenic PDCs ex Vivo

**Figure 7:**
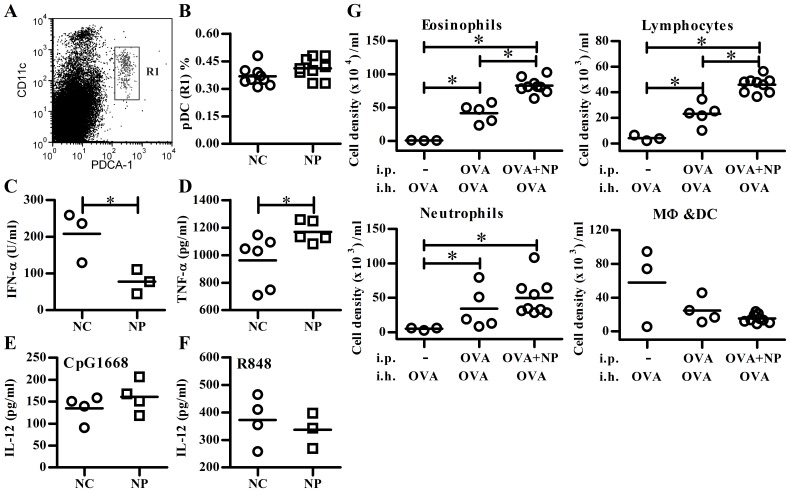
NP administration modulates cytokine production of splenic pDCs and enhances allergic lung inflammation *in vivo*. BALB/c mice were fed daily with nonylphenol (NP) or corn oil only (NC) for 10 days. (**A**) Representative flow cytometric staining pattern of NP-exposed mice with the viable plasmacytoid dendritic cells (pDCs) (R1) is indicated. (**B**) Symbols represent pDC percentage from individual mice in NC or NP-exposed group. Purified pDCs were further stimulated with CpG-D19 for IFN-α (**C**), CpG-1668 for TNF-α (**D**) and IL-12 (**E**) or R848 for IL-12 (**F**) production. Each symbol represents data from individual mice (**B**–**D**) or pool cells from two mice (**E**, **F**). (**G**) BALB/c mice were intraperitoneally (i.p.) received PBS, OVA or OVA plus NP (50 µg/kg BW) emulsified with Al(OH)_3_ on day 0. Three weeks later, all groups of mice were received three daily 3% OVA aerosol challenges (inhalation; i.h.). The next day after the last challenge, cells in bronchoalveolar lavage fluid were analyzed using flow cytometry. *N* = 3–9 mice per group. Data are from two independent experiments. The line within the vertical points marks the mean for each group. *: *p<*0.05 compared with NC using nonparametric Mann-Whitney *U* test. DC, dendritic cells; MΦ, macrophages.

To further examine the *in vivo* effect of NP on pDCs, BALB/c mice were fed with 5 µg NP/kg BW for 10 days daily. This dosage is based on human tolerable daily intakes (5 µg/kg BW) of NP, determined by the Danish Environmental Agency [23]. First, we analyzed the percentages of splenic pDCs (CD11c^low^PDCA-1^+^) (R1 gate in Fig. 7A) in NP-exposed mice. There was no obvious effect of NP on the numbers and percentages of pDCs (Fig. 7B). Next, splenic pDCs from NP-exposed mice were purified and stimulated with TLR agonists to assess their function. As shown in Figs. 7C and 7D, pDC from NP-exposed mice secreted less IFN-α and more TNF-α than the control group; however, NP exposure did not affect IL-12 production in pDCs in response to either CpG1668 (TLR9) (Fig. 7E) or R848 (TLR7 or/and TLR8) (Fig. 7F). These data demonstrate the *ex vivo* effect of NP on pDCs’ function.

### NP Administration Enhanced the Allergic Lung Inflammation in OVA-Induced Model in Vivo

Since pDCs exposed to NP switched their cytokine pattern from type I IFNs and IL-10 to TNF-α, it suggested that NP exposure might enhance the allergic responses of diseases such as asthma. We then examined the effect of NP on an OVA-induced asthma model. As shown in Fig. 7G, NP administration significantly increased the numbers of eosinophils and lymphocytes in the bronchoalveolar lavage fluid of OVA-sensitized and challenged BALB/c mice. This suggests that NP exposure could, at least in part, alter pDC function and is associated with the enhanced allergic responses.

## Discussion

This is the first report, to our knowledge, to demonstrate that alkylphenols alter the functions of pDCs *in vitro* and *in vivo*. Alkylphenols are virtually ubiquitous in our environment and have weak estrogenic activity. Due to their lipophilic properties, they tend to bio-accumulate in human tissues and potentially disturb the endocrine and immune systems. In the present study, NP and 4-OP did suppress the production of type I IFNs and IL-10, but they were shown to enhance TNF-α production in human pDCs *in vitro*. Also, pDCs from NP-exposed mice decreased the type I IFN response but enhanced the proinflammatory cytokine production *ex vivo*. Our data show that NP may inhibit type I IFN responses of pDCs by downregulating IRF-7 and ERK activity and lead to decreased anti-viral activity. In addition, NP may promote TNF-α production by enhancing MKK3/6-p38 MAPK pathway and mediating histone acetylation at the *TNFA* gene locus. These results illustrate the potential impact of environmental hormones on immune regulation, and provide a basis for ultimately establishing their causal relationship with the development of inflammatory diseases.

In our study, the experimental concentrations of alkylphenols used were in the range of reported body burden. Tissue concentrations of NP have been measured in the 1–20 µM range in aquatic organisms [24]. The plasma concentration of NP in New Zealanders has been measured at about 0.32 ug/L [25]; which is between 10^−9^ M and 10^−8^ M. In addition, due to the lipophilic property, the level of NP in adipose tissue is 10-fold higher than that in plasma [19]. In the present study, the effective concentration of alkylphenols on cytokine?expression of human pDCs was between 10^−9^ and 10^−7^ M. We also simulated the human exposure in the *in vivo* experiment. Based on human tolerable daily intakes of NP determined by the Danish Environmental Agency [23], mice had oral exposure to 5 µg/kg BW/day of NP for 10 days in order to mimic the route of human exposure. Although in comparison to the human long-life exposure, our murine experiment was still a relatively short-term NP exposure, splenic pDCs purified from NP-exposed mice still displayed a dysregulated cytokine pattern similar to that observed in the human data.

Our *in vivo* experiments (Fig. 7G) showed evidence supporting that alkylphenol exposure may enhance the allergic inflammation in the context of OVA-induced asthma model. This effect may be associated with, at least in part, the altered cytokine pattern in NP-conditioned pDCs. However, it is, at present, unclear as to whether the increased allergic response is directly as the result of NP effect in pDCs. The fact that NP may be the potential ligand for both ERs and aryl hydrocarbon receptor (AhR) may complicate the *in vivo* effect of NP. Moreover, various cell types are known to be regulated by ERs or AhR, including mucosal epithelial cells, myeloid DCs, innate lymphoid cells, Th17 cells as well as pDCs [26]–[28], which all can be potentially targeted by alkylphenols and contribute to the expression of airway inflammatory responses. Our recent study supports this contention that NP exposure conditions conventional DCs toward Th2-prone phenotype, at least in part, through AhR pathway [17]. In this context, the combined effect of alkylphenol-conditioned pDC and conventional DC may contribute to the pathogenesis of allergic inflammation. The relative contribution of each cell type modulated as the result of alkylphenol exposure *in vivo* needs to be investigated more comprehensively and in depth.

Our study also suggests that exposure to alkylpenols may disturb the immune response to viral infection. The IRF family controls DC activity and also type I IFN induction in DCs. IRF-7 and IRF-3, activated upon TLR signaling, are required for IFN-α and IFN-β induction in pDCs [11]. Some viral infections can interfere with type I IFN induction via inhibition of IRF activation. For example, RV14 infection inhibits the host type I IFN response by interfering with IRF-3 activation [29]. CpG DNA activates the MAPK and IRF-7 pathway and further leads to the expression of IFN-α in human pDCs [30]. These findings, together with the suppressive effect of NP on the MEK1/2-ERK-Elk-1 pathway and IRF-7, the master transcriptional proteins in the development of the host antiviral response, raise a strong possibility that exposure to alkylphenols may impair the ability to eliminate the viral particle via suppression of IRF-7 expression.

It has been shown that human pDCs produce IFN-α through p38 MAPK dependent STAT1 phosphorylation in response to CpG [31]. This study did not address the role of ERK and JNK pathway in CpG-induced IFN-α of pDCs. Our study clearly shows that these three members of the MAPK family are all involved in CpG-induced IFN-α production in human pDCs. In addition, NP inhibited ERK, but not the other two pathways, and down-regulated both IFN-α and IFN-β production in pDCs. However, further study is needed to address whether ERK directly controls IRF-7 expression and how NP disturbs type I IFN production in pDCs.

The finding that ICI182,780 (at relative high concentration, 10^−5^ M) could reverse, at least in part, NP-induced TNF-α and IFN-β?expression?suggests the involvement of the ERs in mediating NP’s effect. However, ICI182,780 was unable to reverse the suppressive effect of NP on IL-10 or IFN-α expression in pDCs. In addition, the effect of E2 on TNF-α and IL-10 expression is not occurring at a physiological dose. These data suggest that there are other unidentified (nuclear) receptors involved in NP’s effect in addition to ERs. The possibility that the involved nuclear receptors are the AhR and the androgen receptor is supported by studies using luciferase reporter gene assays showing that NP may be the potential ligand for AhR and androgen receptor [32]. Evidence also shows that E2 binds to androgen receptor at concentrations around 100 times higher than the levels required to occupy ER and induce responses [33]. However, AhR antagonists did not reverse the suppressive effect of NP on IL-10 or IFN-α expression in human pDCs (data not shown). Therefore, these results suggest the existence of a complex regulatory pathway of alkylphenols involved in cytokine regulation in pDCs with ER dependent and independent pathways, and the involved nuclear receptor(s) and detailed signaling events associated with differential cytokine responses await further investigation.

Epigenetic modifications of the *TNFA* locus have been reported to occur both developmentally and in response to acute stimulation. It has been reported that these modifications actively regulate TNF-α expression in monocytes [15], [16]. Acetylation of core histones allows the chromatin structure to transform into an activated open form and then allows the binding of TATA box-binding protein (TBP), TBP-associated factors and RNA polymerase II, which initiates gene transcription [34]. In our recent study, we demonstrated that NP may induce TNF-α production through histone modification of the *TNFA* gene in myeloid DCs [35]. In this study we also demonstrated that NP-treated pDCs secrete more TNF-α through differential histone acetylation of the *TNFA* gene.

Epigenetic changes such as histone acetylation and DNA methylation are associated with immunological diseases. Emerging evidence suggests that environment-epigenetics mediated immune dysregulation contribute to asthma pathogenesis. Ambient air pollution is associated with hypermethylation of *FOXP3* in regulatory T cells, impairing their suppressive function and increasing asthma morbidity [36]. In addition, loss of DNA methylation and gain of H3 acetylation in IL-4 and IFN-γ genes regulate T-cell lineage differentiation [37], [38]. Thus, changes in epigenetic marks may represent an important pathway by which environmental factors influence disease susceptibility in humans. However, the detailed mechanism awaits further investigation.

In summary, we have demonstrated a previously uncharacterized role of EDCs – alkylphenols in disturbing immune functions of pDCs in human and in mice. We have also clarified that alkylphenol exposure conditions pDCs toward an inflammatory pattern, possibly due to the abilities to impact on the IRF-7, MAPK pathway and epigenetic regulation. Our findings provide a potential link between environmental chemicals and dysregulation of the immune system, and provide a basis for further investigating the precise mechanisms of how alkylphenols affect the function of pDCs.

## Supporting Information

Figure S1 **4-OP stimulates TNF-α but suppresses IL-10 production of human pDCs.** 4-OP treated with purified human pDCs as the same culture conditions as that in Fig. 1 and Fig. 3A. Data show means ± SD of five independent experiments. *p<0.05 was considered significant versus control.
(TIF)

Figure S2 **4-OP significantly suppresses CpG-induced Type I IFN production and in human pDCs.** Purified pDCs were treated with 4-OP for two hours and then stimulated with CpG plus IL-3 for 24 or 48 hours. The supernatant was collected for IFN-α (A) and IFN-β (B) detection using ELISA method. Results show means ± SD of five independent experiments. * or #, *p*<0.05 was considered significant versus corresponding untreated cells.
(TIF)

Figure S3 **NP inhibits CpG-induced IRF-7 expression and decreases transcription of the IFNA genes in human pDCs.** (A) Purified human pDCs were pre-treated with NP at various concentrations for 2 h and then stimulated with CpG plus IL-3 for another 1 h for IRF-3 or 6 h for IRF-7 expression. The total cell lysate was analyzed by western blotting. (B) The expression of IRF7 and IFNA genes in pDCs. Shown is RT-PCR analysis of different treated conditions as described in (A). Data are from one representative of three independent experiments.
(TIF)
