# Supplementary figures and images for: Environmental Alkylphenols Modulate Cytokine Expression in Plasmacytoid Dendritic Cells
Source: PLoS One. 2013 Sep 11;8(9):e73534. doi: 10.1371/journal.pone.0073534 (PMC3770601; doi:10.1371/journal.pone.0073534)

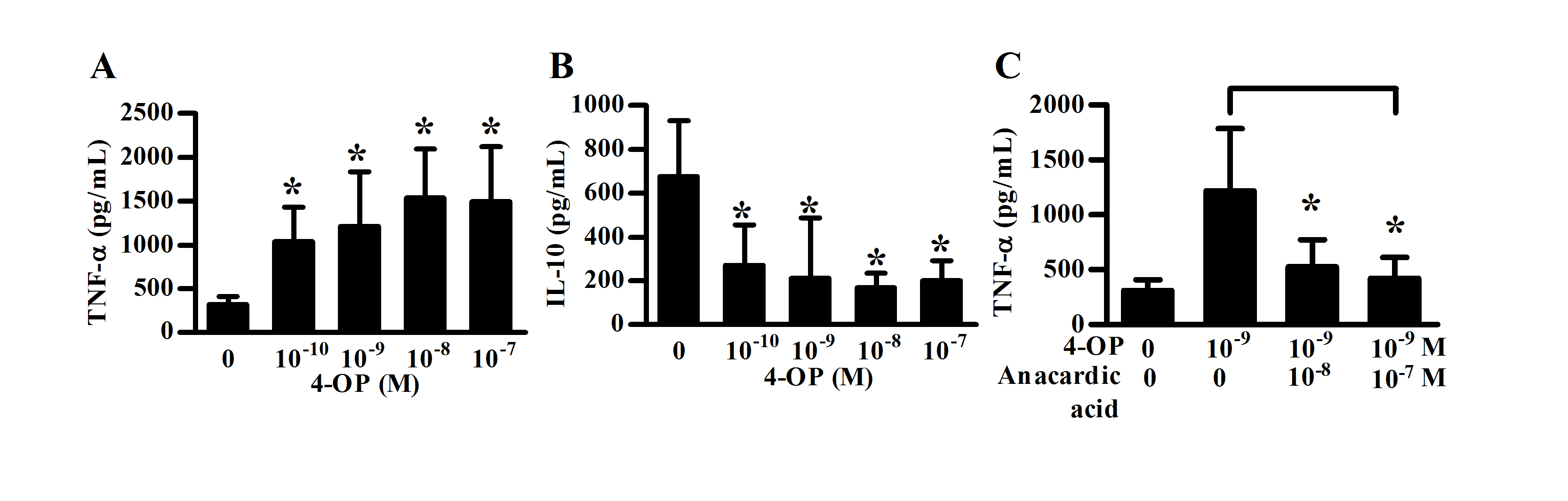

Supplement: Figure S1 — 4-OP stimulates TNF-α but suppresses IL-10 production of human pDCs. 4-OP treated with purified human pDCs as the same culture conditions as that in Fig. 1 and Fig. 3A. Data show means ± SD of five independent experiments. *p<0.05 was considered significant versus control. (TIF) [file pone.0073534.s001.tif]

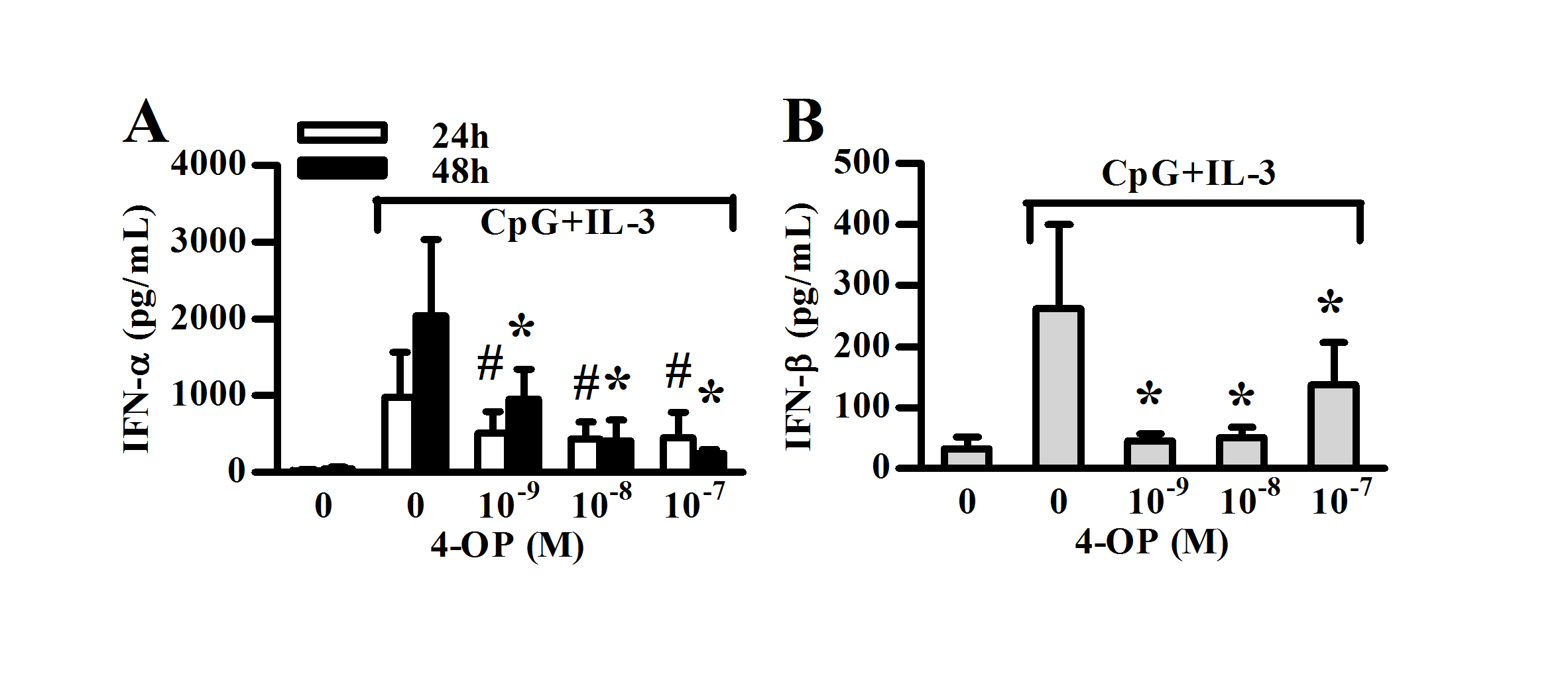

Supplement: Figure S2 — 4-OP significantly suppresses CpG-induced Type I IFN production and in human pDCs. Purified pDCs were treated with 4-OP for two hours and then stimulated with CpG plus IL-3 for 24 or 48 hours. The supernatant was collected for IFN-α (A) and IFN-β (B) detection using ELISA method. Results show means ± SD of five independent experiments. * or #, p<0.05 was considered significant versus corresponding untreated cells. (TIF) [file pone.0073534.s002.tif]

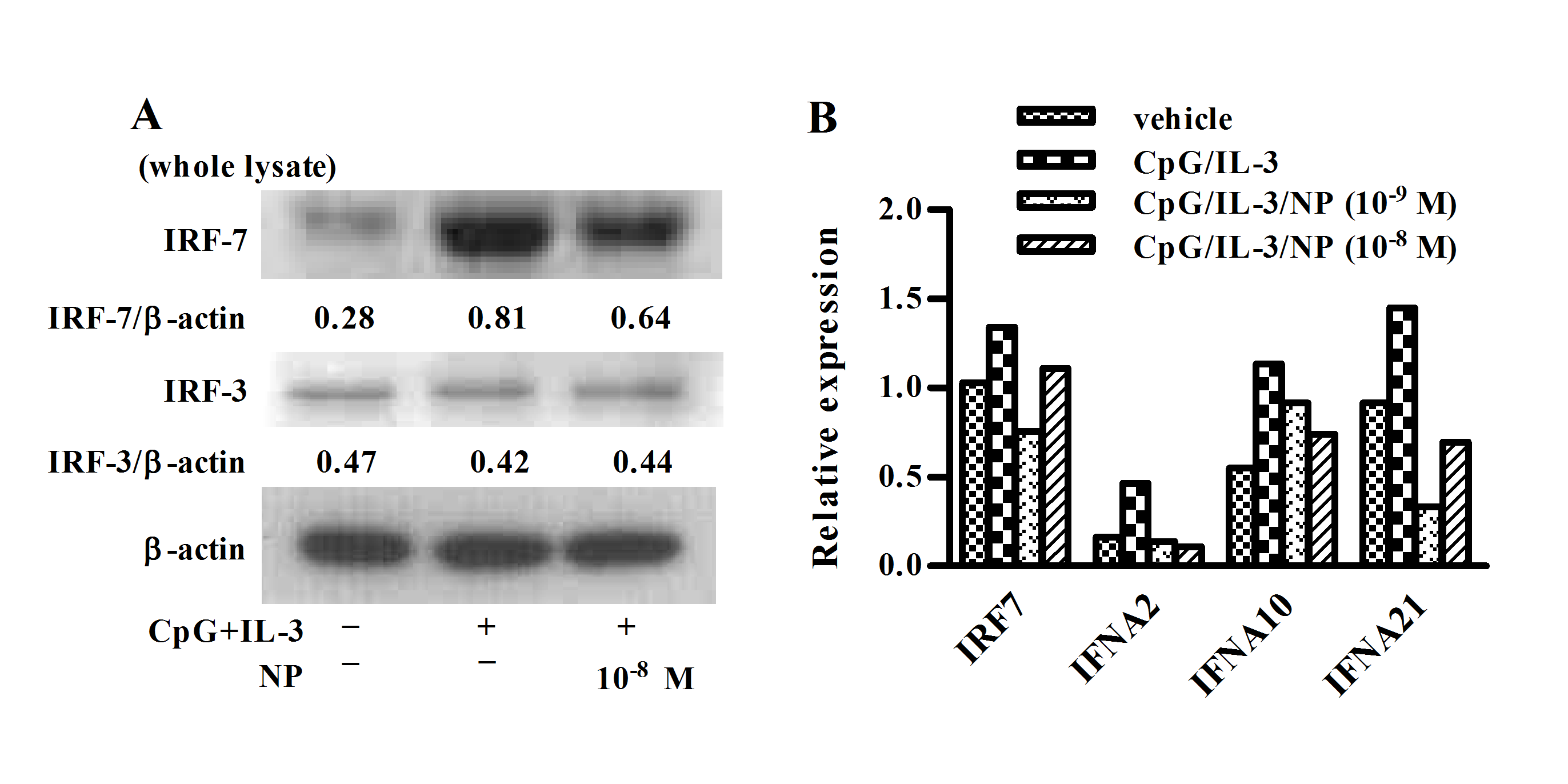

Supplement: Figure S3 — NP inhibits CpG-induced IRF-7 expression and decreases transcription of the IFNA genes in human pDCs. (A) Purified human pDCs were pre-treated with NP at various concentrations for 2 h and then stimulated with CpG plus IL-3 for another 1 h for IRF-3 or 6 h for IRF-7 expression. The total cell lysate was analyzed by western blotting. (B) The expression of IRF7 and IFNA genes in pDCs. Shown is RT-PCR analysis of different treated conditions as described in (A). Data are from one representative of three independent experiments. (TIF) [file pone.0073534.s003.tif]
